# Prognosis prediction in traumatic brain injury patients using machine learning algorithms

**DOI:** 10.1038/s41598-023-28188-w

**Published:** 2023-01-18

**Authors:** Hosseinali Khalili, Maziyar Rismani, Mohammad Ali Nematollahi, Mohammad Sadegh Masoudi, Arefeh Asadollahi, Reza Taheri, Hossein Pourmontaseri, Adib Valibeygi, Mohamad Roshanzamir, Roohallah Alizadehsani, Amin Niakan, Aref Andishgar, Sheikh Mohammed Shariful Islam, U. Rajendra Acharya

**Affiliations:** 1 Trauma Research Center, Shahid Rajaee (Emtiaz) Trauma Hospital, Department of Neurosurgery, Shiraz University of Medical Sciences, Shiraz, Iran; 2 Student Research Committee, Fasa University of Medical Sciences, Fasa, Iran; 3 Department of Computer Sciences, Fasa University, Fasa, Iran; 4 Noncommunicable Diseases Research Center, Fasa University of Medical Sciences, Fasa, Iran; 5 Bitab Knowledge Enterprise, Fasa University of Medical Sciences, Fasa, Iran; 6 Department of Computer Engineering, Faculty of Engineering, Fasa University, Fasa, 74617-81189 Iran; 7 Institute for Intelligent Systems Research and Innovation (IISRI), Deakin University, Geelong, Australia; 8 Institute for Physical Activity and Nutrition, School of Exercise and Nutrition Sciences, Deakin University, Geelong, VIC Australia; 9 Cardiovascular Division, The George Institute for Global Health, Newtown, Australia; 10 Sydney Medical School, University of Sydney, Camperdown, Australia; 11 Department of Electronics and Computer Engineering, Ngee Ann Polytechnic, Singapore, Singapore; 12 Department of Biomedical Engineering, School of Science and Technology, Singapore University of Social Sciences, Singapore, Singapore; 13 Department of Bioinformatics and Medical Engineering, Asia University, Taichung City, Taiwan

**Keywords:** Computational biology and bioinformatics, Medical research, Neurology, Risk factors, Biomedical engineering

## Abstract

Predicting treatment outcomes in traumatic brain injury (TBI) patients is challenging worldwide. The present study aimed to achieve the most accurate machine learning (ML) algorithms to predict the outcomes of TBI treatment by evaluating demographic features, laboratory data, imaging indices, and clinical features. We used data from 3347 patients admitted to a tertiary trauma centre in Iran from 2016 to 2021. After the exclusion of incomplete data, 1653 patients remained. We used ML algorithms such as random forest (RF) and decision tree (DT) with ten-fold cross-validation to develop the best prediction model. Our findings reveal that among different variables included in this study, the motor component of the Glasgow coma scale, the condition of pupils, and the condition of cisterns were the most reliable features for predicting in-hospital mortality, while the patients’ age takes the place of cisterns condition when considering the long-term survival of TBI patients. Also, we found that the RF algorithm is the best model to predict the short-term mortality of TBI patients. However, the generalized linear model (GLM) algorithm showed the best performance (with an accuracy rate of 82.03 ± 2.34) in predicting the long-term survival of patients. Our results showed that using appropriate markers and with further development, ML has the potential to predict TBI patients’ survival in the short- and long-term.

## Introduction

Traumatic brain injury (TBI) is among the most common causes of in-hospital death and neurological disabilities^1^. Recent observations showed that the mortality and morbidity of TBI are growing^2,3^. Over the last two decades, several studies have been dedicated to investigating the risk factors related to TBI morbidity and mortality. For instance, it has been found that age, gender, and the severity of TBI play essential roles in 10-year mortality^4^. Further investigations also introduced multiple risk factors for TBI mortality, such as intracranial pressure (ICP), using alcohol, the intensity of care, oxidative stress imbalance, and grouping complications. Although different risk factors have been distinguished in recent years, we still have a long way to go to achieve accurate assessment scales to manage patients with TBI^5–7^.

Glasgow coma scale (GCS) is a popular tool to assess the neurological condition of patients with different brain injuries, especially TBI. Although GCS provides a reliable measurement for clinicians to manage the TBI, more efficient predictors are required to predict the outcomes of these cases^8^. Glasgow outcome scale (GOS) was another tool recruited to monitor the long-term recovery of patients, which has been extended from 5 to 8 classes (extended GOS or GOSE) to provide a more detailed follow-up^9^. It appropriately depicts the clinical outcomes at discharge and even several months after patient discharge. Recent studies demonstrated that other factors, such as age, the motor component of GCS, pupillary reactivity, and type of injury, significantly influence the prediction of clinical outcomes^10,11^. Recently, novel machine learning (ML) methods have been developed that provide accurate results on medical data such as TBI datasets^12^. Despite achieving promising results on low dimensional problems, ML fails to learn effectively from high dimensional data (e.g. images) due to the curse of dimensionality. Deep learning (DL) models can handles raw high dimensional data. While DL methods are designed to work with high dimensional data, they should be able to work with low dimensional data as well. The only technical consideration that must be taken into account is using simple and shallow deep networks to avoid overfitting and reduce unnecessary computational complexity. However, to compare ML and DL in medical diagnosis, we settled on using a limited but salient feature set in our data collection process.

## Literature review

In 2009, Guler et al.^13^ investigated the application of artificial neural network (ANN) to develop a diagnostic system and determine the severity of TBI. This small study analyzed simple clinical features among 32 cases, including vital signs, GCS, and electroencephalography (EEG), using a 3-layered ANN to find the similarities. This study showed that neurological and systematic features of TBI cases are similar by more than 90%.

Rughani et al.^14^ used 11 clinical inputs to predict hospital survival in individuals with head injury by an ANN and compared it with clinician diagnosis and regression models. The data analysis of 7769 patients showed that ANN models are more accurate, sensitive, and discriminating than clinicians and regression models. The specificity, however, was the same across all models. Although this study showed that ANN would represent a more efficient model for predicting the outcomes of patients with head injuries, there is still a significant gap between the present models and the actual clinical scenarios.

In a study by Shi et al.^5^, ANN was used to develop more accurate predictor models for in-hospital mortality after TBI surgery. The clinical inputs of 16,956 patients were analyzed to compare the performance of ANN and logistic regression (LR) models. Like previous observations, this study showed that ANN model is significantly more accurate, sensitive, and specific. Moreover, the ANN model demonstrated a higher area under the curve (AUC), positive predictive value (PPV), and negative predictive value (NPV). The findings showed that hospital volume, Charlson comorbidity index, length of stay, sex, and age would represent the best prediction of in-hospital mortality after TBI surgery.

Chong et al.^15^ compared the efficiency of ML and LR in predicting TBI. This retrospective case–control study included 39 TBI cases and 156 age-matched controls hospitalized from 2006 to 2014. Then, the performance of ML and LR in the prediction of TBI was compared using receiver operating characteristics (ROC). The findings indicated that analysis of four novel features (involvement in road traffic accidents, loss of consciousness, vomiting, and signs of a base of skull fracture) by ML improved diagnostic parameters (sensitivity (94.9% vs 82.1%), specificity (97.4% vs 92.3%), PPV (90.2% vs 72.7%), NPV (98.7% vs 95.4%), and area under the curve (0.98 vs. 0.93)) in comparison with LR.

In 2015, Lu et al.^16^ investigated the application of ANN in predicting long-term outcomes in TBI cases. This study included different clinical variables, such as GCS (at admission, 7th day, and 14th day), gender, blood sugar, white blood cells, history of diabetes and hypertension, pupil size, diagnosis to predict the 6-month GOS using ANN, Naïve Bayes (NB), DT, and LR. The findings of 128 adult participants showed that ANN has the best performance among different models (AUC of 96.13%, sensitivity of 83.5%, and specificity of 89.73%).

Another study by Beliveau et al.^17^ tried to optimize the prediction models of the one-year functioning of patients with TBI. Using clinical data from 3142 cases, this prospective study increased the diagnostic parameters of AI through novel techniques, including a subset of train and tests. The results indicated that ANN and other models, like LR, generally have high accuracy with the same AUC.

The study by Pourahmad et al.^18^ was another attempt to optimize the predictive models of prediction in TBI patients. The clinical features of 410 cases (including age, gender, CT scan findings, pulse rate, respiratory rate, pupil size, reactivity, and cause of injury) admitted to Shahid Rajaee Hospital with GCS $\le$ 10 were analysed by a 4-layered ANN combined with DT. This hybrid model improved the accuracy (86.3% vs. 82.2%), sensitivity (55.1% vs. 47.6%), specificity (93.6% vs. 91.1%), and AUC (0.705 vs. 0.695) of the prediction of 6-month GOS in patients with TBI.

In 2019, Hale et al.^19^ applied computed tomography (CT) scans in broadly diagnosing TBI. In this study, six clinical features and 17 different variables of CT scan of 480 patients (< 18 years old) were included in an analysis by a two-layer feed-forward ANN with 11 sigmoid hidden and softmax output neurons. The results of this study showed that applying a CT scan to diagnose clinically relevant TBI would significantly increase all diagnostic parameters and achieve a highly optimized predictive model in the future.

A recent study by Abujaber et al.^20^ investigated the application of ML models to predict in-hospital mortality for patients with TBI. The clinical and demographic features of 1620 patients, alongside their CT scan findings, were included in this study to develop efficient models using ANN and support vector machines (SVM). The results showed that SVM is more sensitive (73 vs. 62), accurate (95.6 vs. 91.6), and specific (99 vs. 96) than ANN and has a higher AUC (96 vs. 93.5) and F-score (0.8 vs. 0.64) in predicting the in-hospital mortality.

Recently, Thara et al.^21^ conducted a novel study comparing ML and nomogram performance in predicting intracranial injury in children with TBI. Initially, the clinical parameters of 964 young patients with mild TBI, such as age, sex, road traffic injury, loss of consciousness, amnesia, hemiparesis, scalp injury, bleeding per nose or ear, hypotension, bradycardia, seizure, GCS at emergency department (ED), pupillary light reflex were fed to various classifiers namely SVM, LR, NB, k-nearest neighbors, DT, RF, gradient boosting classifier (GBC), and ANN. The findings showed that RF best predicts pediatric TBI using different clinical features, especially CT scans.

In 2021, Hodel et al.^22^ explored databases such as EBSCOhost CINAHL Complete, PubMed, and IEEE Xplore, to find all publications that developed prediction models for spinal cord injury (SCI). The searches showed that twelve different predictive models were developed in seven unique studies to predict the following clinical outcomes in patients with SCI. This review clearly showed that providing a comprehensive overview of patients with neurological traumas using different ML models would improve our clinical decision-making in the future to make the least mistakes.

Mawdsley et al.^23^ conducted a study to systematically review the efficiency of ML models in predicting different psychosocial aspects of TBI cases. This comprehensive study found nine studies that included eleven types of ML to predict various outcomes. The findings showed that although these models could successfully develop predictive models, there is a lack of evidence to choose ML algorithms as a reliable tool in clinical decision makings.

In 2017, a critical review by Alanazi et al.^24^ evaluated the quality of ML models in predicting patients' outcomes with different disorders. This study showed that AI could provide several promising models to predict these outcomes using patients' multiple clinical, demographical, and imaging data. But, still, we face some limitations in applying these models in clinical situations. Some studies indicated that these novel models would demonstrate significant errors and low efficiency even using the same database. Therefore, further studies are required to increase the reliability of provided models in the future.

In 2022, Choi et al.^25^ developed new models to predict the diagnosis and prognosis of TBI patients at the prehospital stage. This multi-center retrospective study included 1169 TBI cases that were admitted from 2014 to 2018 in different hospitals in Korea. Various features, such as intracranial hemorrhage, admission with/without the ED, and other demographic characteristics, were applied in five ML models, including LR, extreme gradient boosting, SVM, RF, and elastic net (EN). The findings of this study confirmed that EN would significantly develop the overview of the prediction of TBI outcomes at the prehospital stage by increasing AUC, specificity, and sensitivity.

In this year, Daley et al.^26^ tried to provide effective ML-based models to predict severe TBI in admitted patients. This study used neurological and biological data, such as partial thromboplastin time (PTT), motor component of GCS, serum glucose, the fixed pupil(s), platelet count, and creatinine to evaluate the predictive performance of different ML algorithms in the prediction of TBI in 196 admitted children. The findings of this study showed that the optimized models achieve the highest available accuracy (82%) and AUC (0.90).

There are inconsistencies in choosing the best clinical or para-clinical features and the most accurate machine learning model to predict the TBI patients’ outcomes. Hence, the present study is designed to address these problems by recruiting a large population and a wide range of variables using different ML and regression algorithms.

### Dataset description

We used data from 3347 patients in the present study collected from admitted patients at Shahid Rajaee Hospital (Tertiary Trauma Centre), Shiraz, from 2016 to 2021. After the exclusion of patients with incomplete data, 1653 patients remained. The mean ± SD age of the final studied population was 39.55 ± 19.41, which consisted of 1371 men (82.9%). The set of features gathered from the studied patients are available in Table 1.

**Table 1:** Details of our dataset features: the second/third column contains frequency/percent for discrete features and mean/standard-deviation for continuous ones, respectively.

Variable	Frequency or mean	Percent or SD
**Demographic features**		
Gender		
Male	1371	82.9
Female	282	17.1
Smoking	132	8.0
Opium	111	6.7
**Health status**		
Hypertension	124	7.5
Diabetes mellitus	83	5.0
Cardiovascular disease	52	3.2
**Condition of traumatic brain injury**		
Subarachnoid Hemorrhage	571	34.5
Intraventricular Hemorrhage	173	10.5
Epidural Hematoma	469	28.4
Subdural Hematoma	509	30.8
Intracerebral Brain Hemorrhage	755	45.7
Decompressive craniectomy	251	15.2
Pneumocephalus	285	17.2
Base skull fracture	667	40.4
**Cisterns**		
Compressed	126	7.6
Absent basal Cisterns	188	11.4
Midline shift > 5 mm	273	16.5
Depressed skull fracture	185	11.2
**Clinical features**		
** GCS motor components:**		
No motor response	175	10.6
Extensor response	75	4.5
Abnormal flexion	88	5.3
Withdraws pain	191	11.6
Purposeful movement to painful stimulus	520	31.5
Obeys commands	604	36.5
** Pupils**		
Anisocoric	105	6.4
Brisk	1192	72.1
Fixed	235	14.2
Sluggish	4	0.2
Unable to check	94	5.7
Bilateral non-reactive	23	1.4
**Lab data**		
INR	1.31	0.48
First blood sugar	170.30	64.15
Fibrinogen level	239.31	82.28
**Outcomes**		
** GOS**		
Dead	Dis*:319, UP6**:396	Dis:19.30, UP6:23.96
Vegetative state	Dis:191, UP6:36	Dis:11.55, UP6:2.18
Severe disability	Dis:252, UP6:101	Dis:15.25, UP6:6.11
Moderate disability	Dis:302, UP6:198	Dis:18.27, UP6:11.98
Good recovery	Dis:589, UP6:922	Dis:35.63, UP6:55.78
** GOSE**		
Dead	Dis:319, UP6:396	Dis:19.30, UP6:23.96
Vegetative state	Dis:191, UP6:36	Dis:11.55, UP6:2.18
Lower severe disability	Dis:74, UP6:30	Dis:4.48, UP6:1.81
Upper severe disability	Dis:178, UP6:71	Dis:10.79, UP6:4.30
Lower moderate disability	Dis:128, UP6:93	Dis:7.74, UP6:5.63
Upper moderate disability	Dis:174, UP6:105	Dis:10.53, UP6:6.35
Lower good recovery	Dis:249, UP6:318	Dis:15.06, UP6:19.24
Upper good recovery	Dis:340, UP6:604	Dis:20.57, UP6:36.54
Mortality	Dis:319, UP6:396	Dis:19.3, UP6:24.0

*Dis stands for Discharge.

**UP6 stands for Up to 6 months.

To use the dataset in this research regarding diagnostic and therapeutic purposes, institutional approval was granted on the grounds of existing datasets**.** Informed consent was obtained from all subjects and/or their legal guardian(s). All methods were compliant with relevant guidelines and regulations. To use data, ethical approval was obtained from Shahid Rajaee Hospital (Tertiary Trauma Centre), Shiraz, Iran.

The demographic features included age, gender, smoking (smoker, non-smoker), opium (addicted, non-addicted), health status, hypertension, diabetes mellitus, and cardiovascular disease by asking the patients while taking history. Also, GCS and pupil condition (anisocoric/brisk/fixed/sluggish/unable to check/bilateral non-reactive) were measured during a physical exam. The laboratory data of patients, including international normalized ratio (INR), blood sugar (BS), and fibrinogen level, were recorded from reported measurements in electronic documents. The Marshall score, subarachnoid hemorrhage (SAH), intraventricular hemorrhage (IVH), epidural hematoma (EDH), subdural hematoma (SDH), intracerebral hemorrhage (ICH), base of skull fracture, depressed skull fracture, and cisterna were evaluated using CT-scan imaging. The GOS (1 = dead/ 2 = vegetative state/ 3 = severe disability/ 4 = moderate recovery/ 5 = good recovery) and GOSE (1 = dead/ 2 = Vegetative State/ 3. Lower Severe Disability/ 4. Upper Severe Disability/ 5 = Lower Moderate Disability/ 6 = Upper Moderate Disability/ 7 = Lower Good Recovery/ 8 = Upper Good Recovery) were measured at the discharge day (GOSE0) and after 6 months (fGOSE) by trained specialists. The validity and equality of the specialist measurements were confirmed in a session to evaluate 10 cases.

## Methodology

**Figure 1:**
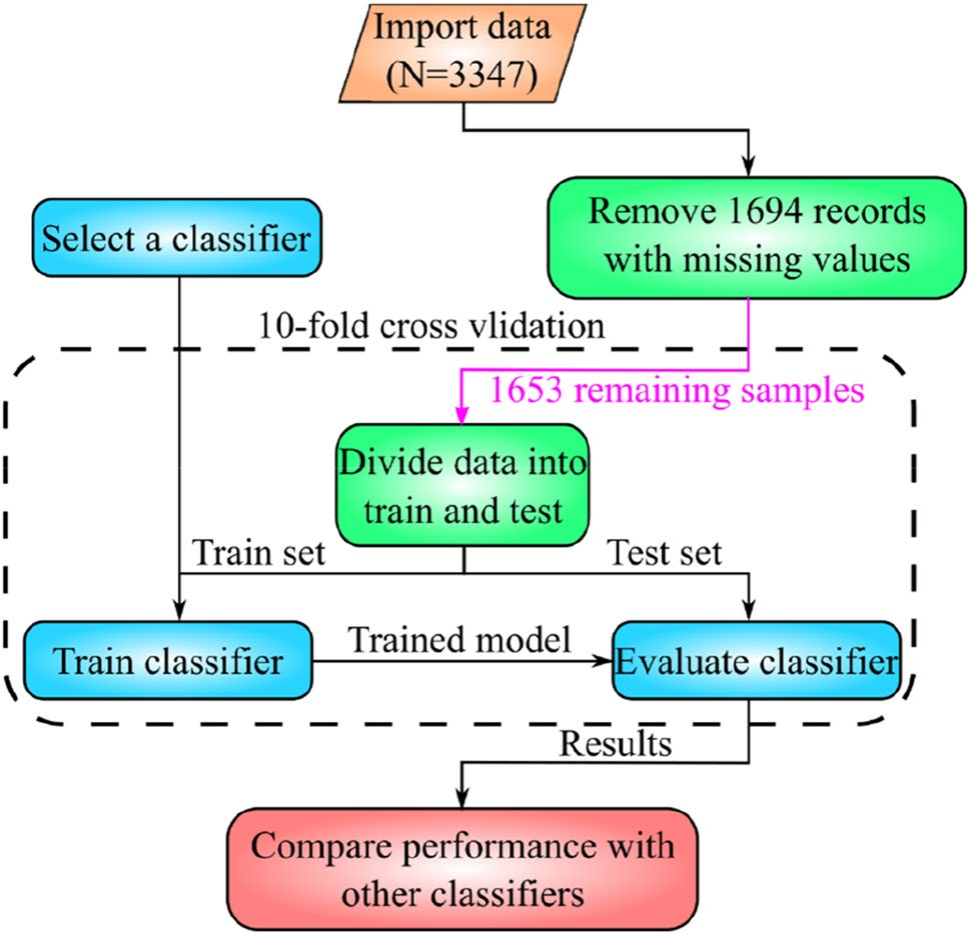
Flowchart of data analysis with different classifier algorithms.

We tested a few state-of-the-art ML algorithms on the dataset according to the flowchart shown in Fig. 1. The target features of our dataset (i.e. the GOS-extended of recovered TBI patients on the GOSE0 and fGOSE) have eight values ({1, 2, …, 8}) that show the level of consciousness. Target feature equal to 1 means no consciousness and the patient dies. On the other hand, when the target feature is 8, the patient can take care of his/her personal affairs. Unfortunately, when the target feature has 8 values (8 classes are defined), the performance of classification algorithms was poor. Therefore, we converted it to a 5-class-dataset according to the physician's suggestion. To this end, classes 3 and 4, 5 and 6, and 7 and 8 were merged. As a result, the performance of classification algorithms was improved significantly.

Given that multiple ML methods have been evaluated during our experiments, they are reviewed briefly in the rest of this section. The presented review will aid with the understanding of the achieved results in the conducted experiments.

### Naïve Bayes (NB)

Naïve Bayes is a probabilistic classifier that is simple yet capable of achieving promising results^27^. The name Naïve Bayes stems from the fact that this method naïvely assumes the features representing input samples are independent. This assumption is not always valid. The classification of input samples is based on the Bayes rule and the parameter estimation is done using maximum likelihood estimation. Suppose $C = \left\{ {C_{1} , \ldots , C_{K} } \right\}$ is the set of possible classes; then the probability that sample $x = \left[ {x_{1} x_{2} \ldots x_{n} } \right]$ belongs to class $C_{k}$ is computed as:

$$ p\left( {C_{k} {|}x} \right) = \frac{1}{Z}p\left( {C_{k} } \right)\mathop \prod \limits_{i = 1}^{n} p(x_{i} |C_{k} ), $$

where Z is called the evident and computed as:

$$ Z = \mathop \sum \limits_{k = 1}^{K} p\left( {C_{k} } \right)p(x|C_{k} ), $$

### Random forest (RF)

**Figure 2:**
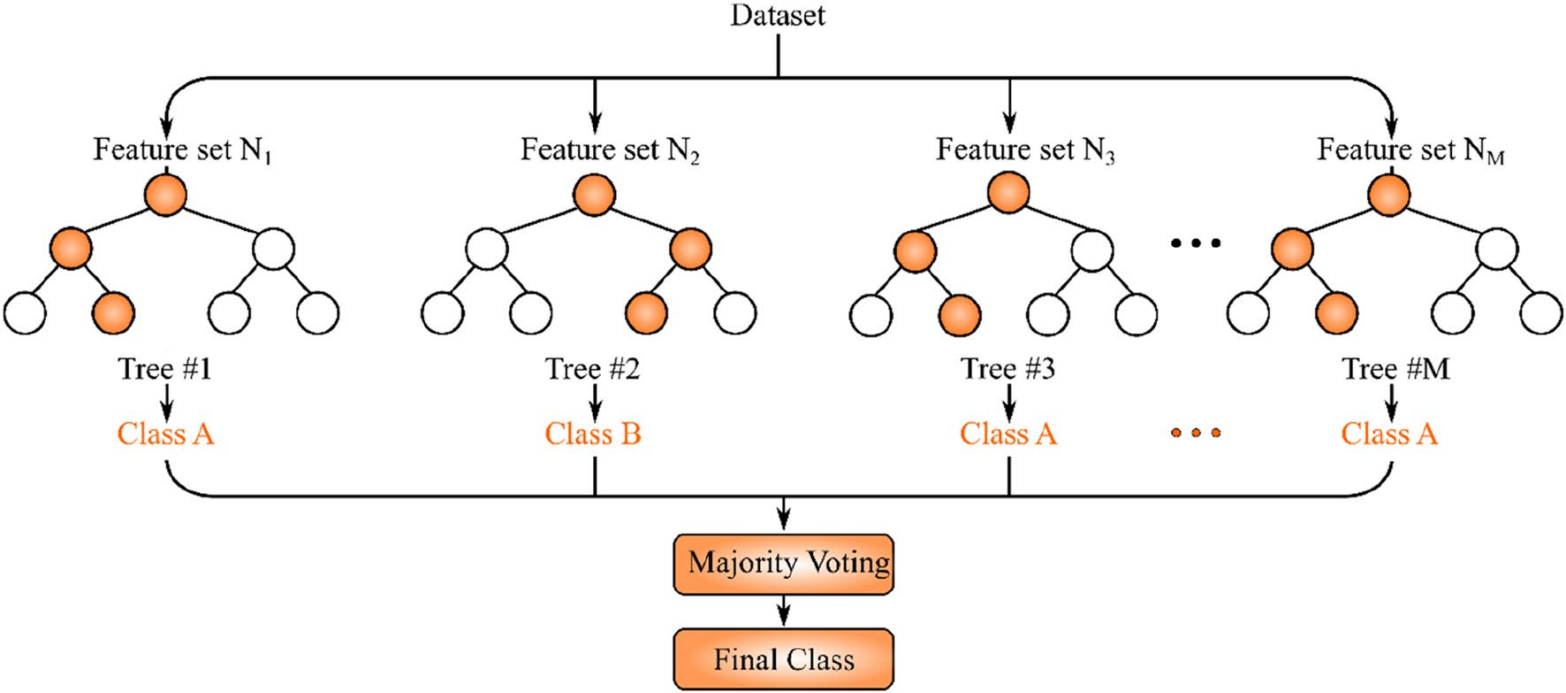
Typical random forest: the final class for each dataset sample is determined by majority voting on decision trees predicted classes.

One of the classic ML methods capable of handling classification and regression is random forest (RF) which is an ensemble approach. As the name implies, RF is made of multiple decision trees each of which consists of multiple decision and leaf nodes. For a classification problem with C classes, the training dataset features are used to create the nodes of the decision trees such that the Gini impurity measure is minimized^28^:

$${I}_{G}\left(n\right)=1-\sum_{i=1}^{C}p{\left(i\right)}^{2},$$

where $p{\left(i\right)}^{2}$ is the probability that a sample from class i is picked in node n. After creating the RF, upon receiving a test sample, it is passed down to each decision tree level by level until it reaches a leaf node. The final step of RF is aggregation of the decision tree outputs. For regression tasks, the aggregation is done by computing the average of the decision tree outputs. For classification tasks, majority voting is performed on the classes predicted by the decision trees to obtain the final output. The schematic of RF inference is shown in Fig. 2. As can be seen, each tree is built using a subset of features of dataset samples. After feeding the input sample to decision trees, majority voting is performed on their predictions to get the predicted class^29^.

### K-nearest-neighbour (KNN)

**Figure 3:**
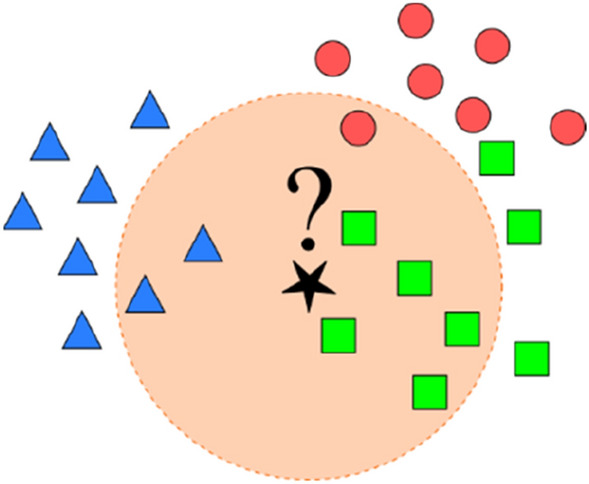
Illustration of KNN (K = 8) classification for test sample (denoted as ?): triangles, squares, and circles represent samples from a 3-class training dataset.

K-nearest neighbour (KNN) is a simple and powerful non-parametric supervised method, which can be used for classification and regression. To classify a test sample, K samples that are closer to the test sample (according to some distance metric) are chosen from the training dataset. In the case of regression, the predicted output for the test sample is computed by taking the average of target values corresponding to K chosen training samples. For classification tasks, the dominant label among the target labels of the K chosen training samples is chosen as the predicted label for the test sample. A typical classification using KNN with K = 8 is shown in Fig. 3. As can be seen, the training dataset contains three classes, the samples of which are shown with triangles, squares, and circles. The test sample is shown with a start. Assuming K = 8, eight nearest neighbours of the test sample are the ones within the neighbourhood circle of the test sample. Given that majority of the eight neighbours are squares, the label of the test sample is predicted as square^30^.

### Rule induction (RI)

One of the ML methods closely related to decision trees is rule induction (RI), which extracts formal rules from observations such that information gain is maximized. The rules are in “if–then” format and are iteratively grown and pruned during the rule extraction process. The advantage of RI is being expressible in first-order logic and ease of encoding prior knowledge in them^31^.

### Deep learning (DL)

**Figure 4:**
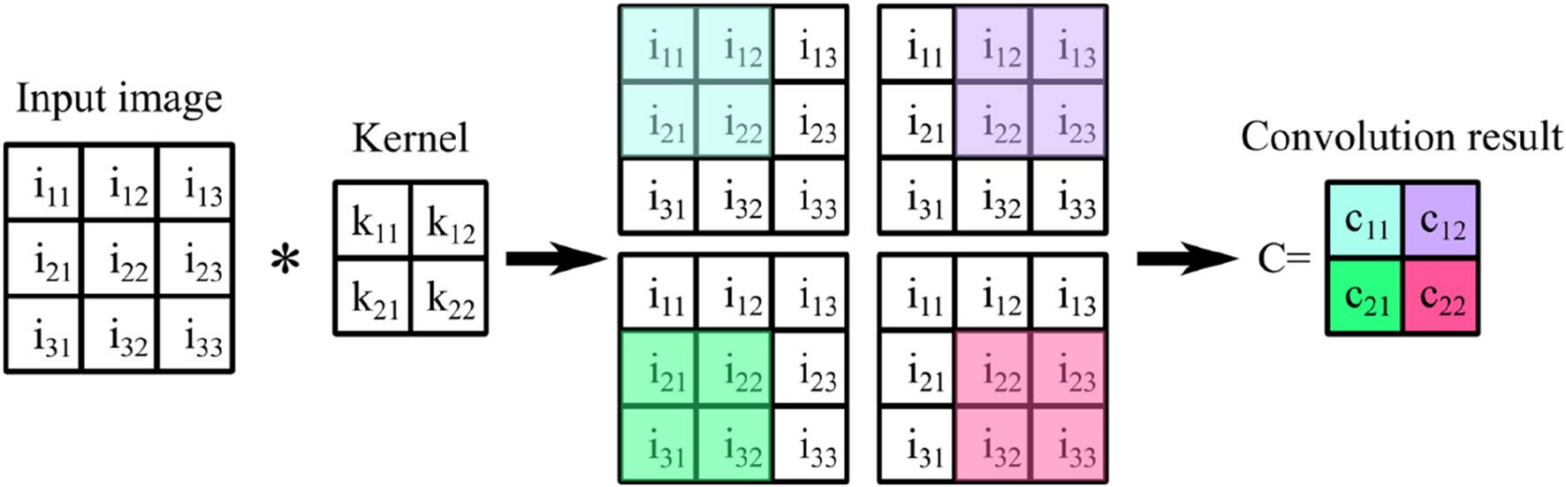
Illustration of convolving a $3\times 3$ input image with a $2\times 2$ kernel.

DL is one of the most promising ML methods capable of efficient feature extraction from high dimensional data. Since the emergence of DL, many challenging high dimensional problems have been solved. The primary building blocks of DL models are trainable filters (kernel) that are convolved with previous layer output (or input sample) to extract salient features depending on the learning problem objective. The process of convolving a typical $2\times 2$ kernel with a $3\times 3$ input image has been depicted in Fig. 4. The kernel is slid on the input image four times to cover all of the image pixels. Each time, the dot product of a subset of image pixels with the kernel is computed. The pixels contributing to the dot product are highlighted in Fig. 4. The output of the convolution is a $2\times 2$ matrix. The colour of each cell ${c}_{ij}$ of matrix $C={\left\{{c}_{ij}\right\}}_{i,j\in \{\mathrm{1,2}\}}$ corresponds to the subset of pixels from the input image that has been used to compute ${c}_{ij}$ value^32^.

### Gradient boosting trees (GBT)

**Figure 5:**
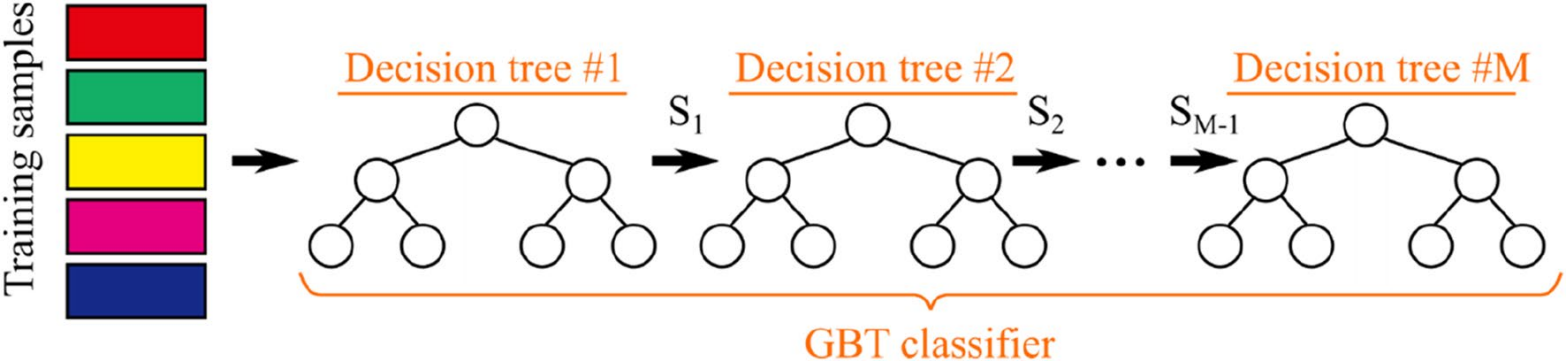
Process of building GBT according to given training set: each ${\mathrm{S}}_{\mathrm{i}}$ is the set of samples misclassified by ith decision tree.

Ensemble learning has proved to be robust and reliable in challenging learning tasks. Gradient Boosting Trees (GBT) employs an ensemble of decision trees (weak learners) to achieve good classification/regression performance while keeping the computational complexity manageable. To this end, decision trees are constrained to be shallow in depth. As shown in Fig. 5, GBT builds the first shallow decision tree using the available training samples. The samples that are misclassified by the first decision tree (set ${S}_{1}$) are then used to build the second tree. The sample set ${S}_{2}$ that has been misclassified by the second decision tree is used to build the third decision tree. The process continues until all of the training samples are classified correctly. The set of built decision trees forms the GBT ensemble classifier. During testing, all decision trees classify the given test samples, and their predictions are aggregated to compute the final output of the GBT^33^.

### K-fold cross-validation

**Figure 6:**
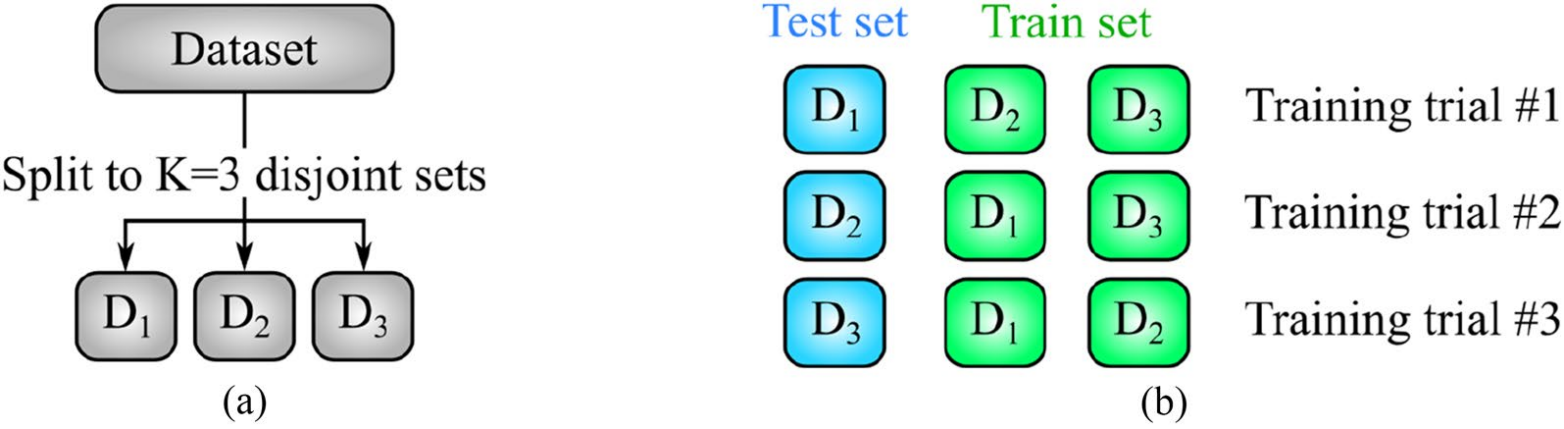
Graphical representation of threefold cross-validation: (a) dataset is partitioned into K = 3 disjoint subsets $\{{\mathrm{D}}_{1},{\mathrm{D}}_{2},{\mathrm{D}}_{3}\}$, and (b) K = 3 training trials. In each trial, one of ${\mathrm{D}}_{1},{\mathrm{D}}_{2}\mathrm{ or }{\mathrm{D}}_{3}$ is used as the test set.

In ML problems, it is customary to split the available dataset into K disjoint subsets with equal sizes and repeat the training process K times. In kth training trial, the kth subset is used for testing and the remaining K-1 subsets are used as training data^34^. As an example, the process of splitting the dataset into K = 3 subsets (also known as folds) is shown in Fig. 6.a. The three subsets $\left\{{D}_{1},{D}_{2},{D}_{3}\right\}$ have no sample in common and are completely disjoint. After splitting the dataset, the training process is repeated K times. In ith training trial, ${D}_{i}$ is used as the test set. The configuration of training and test sets for K = 3 has been shown in Fig. 6.b.

The K training trials yield K values per performance metric. These K values are averaged to report the final performance of ML methods. The motivation behind K-fold cross-validation is the possibility of testing ML methods on all available samples. Moreover, aggregating the performance metrics via averaging leads to a more reliable performance evaluation of the methods mentioned above.

### Performance metrics

In this section, the criteria for comparison of obtained results from the conducted experiments are reviewed. Given the popularity of accuracy, recall (sensitivity), and precision as performance metrics^35–41^, they are used to evaluate the output of our experiments. Accuracy is defined as

1 $$Accuracy=\frac{TP+TN}{TP+TN+FP+FN}$$

where TP (true positive) is the number of positive instances the model correctly predicts as the positive class, TN is the number of negative instances that the model correctly predicts as the negative class, FP (false positives) is the number of negative instances that the model incorrectly predicts as the positive class. Finally, FN (false negative) is the number of positive instances the model incorrectly predicts as the negative class.

The recall for each class of the evaluated dataset is calculated as

2 $$Recall=\frac{TP}{TP+FN}$$

where recall of class ${C}_{i}$ is the fraction of instances that have been indeed classified as ${C}_{i}$. Precision of class ${C}_{i}$ is defined as

3 $$Precision=\frac{TP}{TP+FP}$$

which is the fraction of samples classified as ${C}_{i}$ that indeed belong to the class ${C}_{i}$. AUC indicates the area under the receiver operating characteristic (ROC) curve, and ROC is an evaluation metric for binary classification problems. ROC is the plot of the TP rate vs. FP rate for different threshold values.

## Results

In this section, the obtained results are presented. In all of the remaining tables, the abbreviations Acc, Prec, Rec, and Avg stand for accuracy, precision, recall, and average, respectively. We have applied some of the most important classification algorithms to our patients just when they leave the hospital which has yielded the results in Table 2. The classification algorithms used in this work are NB^42^, RF^43^, KNN(k = 5)^44^, KNN(k = 6), DT^45^, RI^46^, DL^47^ and Gradient Boosting Trees (GBT)^48^ implemented in RapidMiner v9.10^49^. Rapidminer is a comprehensive data science platform with visual workflow design and full automation. It is one of the most popular data science tools. This platform was run on a personal computer with Intel(R) Core(TM) i5-4570, 3.20 GHz processor and 4 GB of RAM.

**Table 2:** The performance of different classification algorithms on 5-class-dataset according to GOS0: Acc, Rec, and Avg stand for accuracy, recall, and average, respectively.

Algorithm	Acc (%)	Acc Rank	Rec1 (%)	Rec2 (%)	Rec3 (%)	Rec4 (%)	Rec5 (%)
NB	44.76 ± 3.94	4	42.95	33.51	11.51	9.60	81.66
RF	45.37 ± 1.53	3	52.66	10.47	1.19	1.66	94.06
KNN(k = 5)	33.82 ± 2.07	8	34.48	9.42	8.33	17.55	60.61
KNN(k = 6)	36.36 ± 2.55	7	40.44	10.99	7.54	17.88	64.18
DT	43.86 ± 1.96	6	53.29	5.24	1.59	1.32	91.17
RI	44.71 ± 3.02	5	44.20	17.80	11.90	8.28	86.42
DL	46.22 ± 1.60	2	55.49	8.90	11.90	11.92	85.57
**GBT**	**47.67 ± 2.65**	**1**	59.25	27.23	8.73	10.60	83.70
Avg Acc	42.85						

According to the obtained result, GBT, DL, and RF have the best accuracy rate of 47.67 ± 2.65, 46.22% ± 1.60%, and 45.37% ± 1.53%, respectively, while KNN (K = 5) has the worst with an accuracy rate of 33.82% ± 2.07%.

As we have more than two classes in this test, only recall for each class was calculated.

Both accuracy and recall of investigated algorithms are shown in Table 2.

Table 3 shows the top 10 features with a higher role in classification and their weights. The weights are calculated by information gain^50^. The GCS motor component on admission (GCSM0), pupil, and Cisterns are the most significant features in classification, respectively.

After six months of leaving the hospital, when the target feature is fGOSE, the patients' conditions were investigated again. As it was shown in Table 4, GBT, RF, and DL have the best accuracy rate of 64.97% ± 1.62%, 64.97% ± 2.72%, and 64.37% ± 1.56%, respectively, while KNN (K = 5) has the worst with an accuracy rate of 55.89% ± 3.72%. As we have more than two classes in this test, only recall for each class was calculated. Therefore, the recall of each class is shown in Table 4.

**Table 3:** Feature weights calculated by information gain applied on 5-classes-dataset of gos0.

Row	Feature	Weight
1	GCSM0	0.1956
2	pupil	0.1265
3	Cisterns	0.0809
4	DC	0.0725
5	Marshall	0.0718
6	age	0.0651
7	INR	0.0425
8	IVH	0.0385
9	1st BS	0.0378
10	Shift	0.0277

GCSM0: motor component of GCS on admission, DC: decompressive craniotomy, INR: international normalized ratio, IVH: intraventricular hemorrhage, BS: blood sugar.

**Table 4:** The performance of different classification algorithms on 5-class-dataset according to fgose.

Algorithm	Acc (%)	Acc rank	Rec1 (%)	Rec2 (%)	Rec3 (%)	Rec4 (%)	Rec5 (%)
NB	59.46 ± 3.22	6	50.51	11.11	0.99	2.02	83.95
RF	64.97 ± 2.72	2	46.21	0.00	0.00	0.00	96.64
KNN(k = 5)	55.89 ± 3.72	8	35.86	0.00	0.99	10.61	82.43
KNN(k = 6)	56.20 ± 1.74	7	35.35	0.00	0.00	9.09	83.62
DT	63.16 ± 1.58	4	55.05	0.00	0.00	1.52	89.26
RI	62.67 ± 2.33	5	47.47	0.00	0.00	5.05	90.89
DL	64.37 ± 1.56	3	55.30	0.00	1.98	3.54	90.67
**GBT**	**64.97 ± 1.62**	**1**	59.85	0.00	0.99	1.01	90.46
Avg Acc	61.46						

In addition, comparing the average accuracy in Table 2 with that of Table 4 shows that predicting the future condition of the patients according to the selected features is more reliable after 6 months.

Table 5 shows the top 10 features with a higher role in classification and their weights. The weights are calculated by information gain. GCSM0, pupil, and age are the most significant features in classification, respectively. Compared to Table 2, the importance of age has increased, and now its role is more important than Cisterns.

**Table 5:** Feature weights calculated by information gain applied on 5-classes-dataset of fGOS.

Row	Feature	Weight
1	GCSM0	0.1144
2	pupil	0.1037
3	age	0.0702
4	Marshall	0.0598
5	Cisterns	0.0580
6	DC	0.0383
7	INR	0.0335
8	1st BS	0.0261
9	IVH	0.0248
10	Shift	0.0237

GCSM0: motor component of GCS on admission, DC: decompressive craniotomy, INR: international normalized ratio, BS: blood sugar, IVH: intraventricular hemorrhage.

We also checked the system's performance when the patients were classified into only two groups, dead and alive. In this case, in addition to the classification mentioned above, two more algorithms LR and GLM were also investigated, which can be applied to only two-class classification problems. In this case, the performance of classification algorithms was again improved compared with the 5-class-dataset. The result of classifying patient into either dead or alive when they leave the hospital are shown in Table 6. Accordingly, the accuracy rates of all algorithms are more than 80% which shows significant improvement compared with classification algorithms applied on the 5-class-dataset. In addition, there is no significant difference between the accuracy rates of most of these algorithms. All algorithms have a performance rate between 80 and 85%. The precision, recall, and AUC are also shown in this table.

**Table 6:** The performance of different classification algorithms on 2-class-dataset according to gose0. Significant values are in bold.

Algorithm	Acc (%)	Acc rank	Prec (%)	Rec (%)	AUC
NB	81.67 ± 1.29	7	52.61% ± 3.42	51.71% ± 7.88	0.820 ± 0.033
RF	**84.45 ± 1.29**	**1**	**76.72% ± 12.75**	27.89% ± 7.10	0.827 ± 0.046
KNN(k = 5)	80.64 ± 2.40	10	50.11% ± 13.99	24.14% ± 7.53	0.659 ± 0.048
KNN(k = 6)	81.07 ± 2.43	9	51.48 ± 13.05	24.13 ± 9.87	0.679 ± 0.056
DT	82.46 ± 1.15	6	59.98 ± 6.95	30.14 ± 8.62	0.703 ± 0.038
RI	83.24 ± 2.94	4	61.84 ± 12.20	39.82 ± 8.62	0.797 ± 0.067
DL	81.13 ± 2.77	8	52.81 ± 8.79	**55.18 ± 12.29**	**0.845 ± 0.029**
GBT	82.82 ± 1.72	5	55.62 ± 3.88	51.72 ± 13.27	0.827 ± 0.046
LR	84.03 ± 1.76	2	64.80 ± 8.94	40.08 ± 8.01	0.842 ± 0.043
GLM	83.91 ± 2.08	3	63.39 ± 9.43	41.08 ± 6.06	0.841 ± 0.039
Avg Acc	82.52				

According to the results shown in Table 6, RF, GLM, and RI have the best accuracy rate, respectively. The confusion matrix of best performing RF classifier is shown in Table 7.

**Table 7:** Confusion matrix obtained using RF classifier.

Actual label	Predicted label	
	Alive	Dead
Alive	1307	27
Dead	230	89

Table 8 shows the top 10 features with a higher role in classification and their weights. The weights are calculated by information gain. Like Tables 3 and 5, the pupil has a significant role in classification. The order of other features does not have a substantial difference between Tables 3 and 5.

**Table 8:** Feature weights calculated by information gain applied on 2-classes-dataset of GOS0.

Row	Feature	Weight
1	Pupil	0.0614
2	Cisterns	0.0560
3	Age	0.0539
4	GCSM0	0.0472
5	Marshall	0.0414
6	INR	0.0345
7	DC	0.0209
8	1st BS	0.0192
9	Shift	0.0186
10	SAH	0.0172

GCSM0: motor component of GCS on admission, INR: international normalized ratio, DC: decompressive craniotomy, BS: blood sugar, SAH: subarachnoid hemorrhage.

The results of applying the classification algorithms on the 2-class-dataset after six months of leaving the hospital are shown in Tables 9. Table 10, 11 shows the importance of the features in classification. Comparing the average accuracy in Tables 6 and 9 shows that the accuracy rate does not change significantly after six months of the patient’s discharge. Finally, the confusion matrix of the best-performing GLM algorithm is shown in Table 10.

**Table 9:** The performance of different classification algorithms on 2-class-dataset according to fGOS. Significant values are in bold.

Algorithm	Acc (%)	Acc Rank	Prec (%)	Rec (%)	AUC
NB	78.65 ± 3.93	7	55.95 ± 8.59	53.82 ± 8.36	0.812 ± 0.039
RF	80.88 ± 1.86	3	**77.32 ± 10.90**	29.04 ± 4.50	0.807 ± 0.035
KNN(k = 5)	**76.95 ± 1.66**	9	54.49 ± 7.76	28.54 ± 2.95	0.661 ± 0.060
KNN(k = 6)	76.95 ± 1.96	10	53.56 ± 7.68	29.27 ± 7.31	0.675 ± 0.052
DT	78.22 ± 1.23	8	63.26 ± 9.17	25.33 ± 8.72	0.683 ± 0.039
RI	80.70 ± 2.51	4	66.69 ± 9.83	41.18 ± 7.83	0.758 ± 0.054
DL	78.95 ± 2.74	6	55.82 ± 5.73	**59.37 ± 10.79**	0.821 ± 0.038
GBT	79.25 ± 3.02	5	57.51 ± 6.86	56.56 ± 7.68	**0.823 ± 0.015**
LR	81.61 ± 2.58	2	67.61 ± 8.10	45.99 ± 4.21	**0.834 ± 0.031**
GLM	**82.03 ± 2.34**	**1**	68.00 ± 6.36	47.22 ± 8.41	0.834 ± 0.038
Avg Acc	79.42

**Table 10:** Confusion matrix obtained using GLM algorithm according to fGOS.

Actual label	Predicted label	
	Alive	Dead
Alive	1169	88
dead	209	187

**Table 11:** Feature weights calculated by information gain applied on 2-classes-dataset of fGOS.

Row	Feature	Weight
1	Age	0.0631
2	Pupil	0.0611
3	GCSM0	0.0547
4	Cisterns	0.0491
5	Marshall	0.0402
6	INR	0.0302
7	DC	0.0232
8	SAH	0.0196
9	1st BS	0.0185
10	IVH	0.0173

GCSM0: motor component of GCS on admission, INR: international normalized ratio, DC: decompressive craniotomy, SAH: subarachnoid hemorrhage, BS: blood sugar, IVH: intraventricular hemorrhage.

Overall, according to the results shown in Tables 2 and 4, GBT has the best performance. RFs and DL are in the next ranks. Meanwhile, the ranks of accuracy in Tables 6 and 9 show that GLM, LR, and RF have better performance than other compared algorithms in the classification of these data. Finally, it should be noted that DL has the best Recall among all of the investigated algorithms in both Tables 6 and 9. The rank-based analysis of investigated algorithms is shown in Table 12.

**Table 12:** Rank-based analysis of investigated algorithms: avg stands for average.

Algorithm	5 Classes			2 Classes			Overall ranks avg
	gos0 rank	fgos rank	Ranks avg	gos0 rank	fgos rank	Ranks avg	
NB	4	6	5	7	7	7	6
RF	3	2	2.5	1	3	2	2.25
KNN(k = 5)	8	8	8	10	9	9.5	8.75
KNN(k = 6)	7	7	7	9	10	9.5	8.25
DT	6	4	5	6	8	7	6
RI	5	5	5	4	4	4	4.5
DL	2	3	2.5	8	6	7	4.75
GBT	1	1	1	5	5	5	3
LR	–-	–-	–-	2	2	2	2
GLM	–-	–-	–-	3	1	2	2

## Discussion

The present longitudinal study primarily aimed to predict the GOS of recovered TBI patients at discharge and six months after discharge. Our findings showed that different machine learning algorithms applied in this study provide acceptable performance using collected health status, demographic features, clinical physical exams, and laboratory data.

The first steps of prediction begin with classifying TBI cases' severity by baseline features. There have been controversies about ML ability to outperform human neurologists. It has been previously claimed that ML algorithms were not more efficient than neurologists^13^. However, Rughani et al. showed that ANN can outperform regression models and clinicians' categorizations regarding survival prediction of TBI patients achieving accuracy of 73%^14^.

The first aim of this paper was to find the most reliable prognostic markers related to TBI. Several features have been introduced as the most reliable variables in recent years. Shi et al. achieved acceptable predictive DL models for in-hospital mortality in patients with TBI based on clinical and demographic features such as gender, age, and Charlson comorbidity index^5^. Other features including vomiting, signs of a skull base fracture, loss of consciousness (LOC), and history of traffic accidents have been introduced as well^15^. However, our assessments on wide background, clinical, and paraclinical features with various models indicated that the condition of pupils, the condition of cisterns (being present, absent, or compressed), and the patient’s age are the best predictors of in-hospital mortality, while the condition of the pupils, GCSM, and age are the most important clinical features in predicting the long-term mortality^51^. Some factors may stand for different findings among the studies, such as entering different variables into the analysis. For instance, we utilized the motor component of GCS rather than the total GCS, which is broadly used in various trials^16^. Supporting our findings, previous studies confirmed that using the motor component of GCS would provide more accurate models than the total GCS^26^.

The second aim of the present study was to provide efficient ML and statistical models to predict the short- and long-term outcomes of TBI patients. The outcomes of TBI would be appropriately predicted using the clinical features of the first day of admission^9^, as discussed earlier. The first evaluations emphasized that all prediction models, based on ML or LR would achieve a high success rate^17^. According to our findings, the RF, LR, and GLM models are the most accurate models to predict the in-hospital mortality of patients (based on the 2-class GOS).

On the other hand, GLM (with an accuracy of 82%) was found to be the most accurate predictor of 6-months mortality. Instead, when using 5-class GOS, GBT was the most accurate predictor of both in-hospital and 6-months follow-up morbidity and mortality. However, as described in the results, the accuracy of the 5-class GOS is lower than the 2-class GOS. Matsuo et al. found that RF is the best model for predicting in-hospital outcomes following TBI which supports our results^52^. Lu et al. conducted a study to compare the efficacy of different ML models and LR in predicting 6-month GOS. ANN showed the best performance using clinical features, with AUC of 0.96^16^.

Applying CT scans in prediction models based on ANN achieved promising outcomes in forecasting the TBI prognosis^19^. As an example, Abujaber et al. employed CT scans as part of their feature set and reported SVM as the best method for in-hospital mortality prediction of TBI patients^20^. In a similar attempt, Steyerberg et al. introduced the Marshal score (a CT scan index) as a major feature of predicting TBI outcomes, alongside glucose, hemoglobin, hypotension, and hypoxia^10^.

The race toward achieving reliable ML model for robust clinical decision-making continues^53^. For example, Lang et al. provided clinical decision support for TBI patients capable of reducing the 7-day mortality showing the ML potential in clinical decision makings^54^. On the contrary, ML failed to outperform LR in predicting the outcome of a large database of patients with moderate to severe TBI^55^. As a result, it has been suggested that the main focus must be on including valuable prognostic markers instead of ML algorithms. Using a more limited number of features and lacking serologic markers, Bruschetta et al.^56^ also reported that LR and ML may have similar performance. Finally, Kazim et al.^57^ reported that ML performance is similar to correlation and multiple linear regression analysis. However, the reported results were based on only 168 patients with severe TBI. In order to present our contribution compared to the ML-based TBI diagnosis methods reviewed above, they have been summarized in Table 13.

**Table 13:** Comparison of our work with the existing ML methods developed for automated detection of TBI.

Authors	Method	Study objective	Dataset features	Results
Guler et al.^13^	ANN	TBI severity assessment	vital signs, GCS, EEG	90% similarity between neurological and systematic features of TBI
Rughani et al.^14^	ANN	Survival prediction of traumatic brain injury	National Trauma Database, 11 clinical features	Better accuracy and sensitivity compared to clinicians and regression
Shi et al.^5^	ANN	Post TBI surgery mortality prediction	hospital volume, Charlson comorbidity index, length of stay, sex, age	Better accuracy, sensitivity, and specificity of ANN over LR
Chong et al.^15^	ML vs LR	TBI prediction	Road accidents, consciousness loss, vomiting, skull fracture	Better Sensitivity (94.9%), specificity (97.4%), AUC (0.98) of ML compared to RL
Lu et al.^16^	ANN, NB, DT, LR	TBI cases long-term outcomes	GCS, gender, blood sugar, white blood cells, diabetes and hypertension history, pupil size	Better AUC (96.13%), sensitivity (83.5%), specificity (89.73%) of ANN
Beliveau et al.^17^	ANN, LR	1-year functioning of TBI patients	National SCIMS Database	Reported good accuracy for ANN and LR
Pourahmad et al.^18^	ANN + DT	6-month GOS prediction in TBI patients	Clinical features (age, gender, CT scan, pulse rate, respiratory rate, pupil size, reactivity, cause of injury)	Improved accuracy (86.3%), sensitivity (55.1%), specificity (93.6%), AUC (0.705)
Hale et al.^19^	ANN	Detecting clinically relevant TBI (CRTBI) patients	6 clinical features + 17 variables of CT scan	Highly optimized predictive model for CRTBI diagnosis using CT scan
Abujaber et al.^20^	ANN, SVM	Predicting in-hospital mortality of TBI patients	Patients’ demographic features and CT scan	SVM outperforms ANN: sensitivity (73% vs. 62%), accuracy (95.6% vs. 91.6%), specificity (99% vs. 96%)
Thara et al.^21^	SVM, LR, NB, KNN, DT, RFC, GBC, ANN	Intracranial injury prediction in children suffering TBI	Clinical features (age, gender, road traffic injury, loss of consciousness, amnesia, etc.)	RFC represents the best performance
Choi et al.^25^	LR, GBC, SVM, RF, elastic net (EN)	Diagnosis/ prognosis of TBI patients at the prehospital stage	Intracranial hemorrhage, admission with (out) emergency department, other demographic characteristics	Increased AUC, specificity, sensitivity using EN
Daley et al.^26^	RF + feature selection	predict severe TBI in admitted patients	Neurological/ biological data (partial thromboplastin time, serum glucose, creatinine, etc.)	Achieved 82% accuracy, 0.9 AUC
Our works	NB, RF, KNN, DT, RL, DL, GBT	TBI survival prediction	Demographic features, health status, traumatic brain injury condition	Several results for 2-class and 5-class problems, salient features determination

The novelties of our proposed model are as follows:

1. We have obtained high performance using simple ML algorithms.
2. Employed large number of patients and used more features compared to existing literature.
3. We have gathered a TBI dataset in Iran.
4. New features such as INR, Fibrinogen level, and CVD/CVA, have been investigated that have not been considered in previous studies.
5. Benchmarking well-known classic ML methods (NB, RF, KNN, DT, RI, GBT) as well as DL on TBI survival prediction.
6. The collected dataset has been analysed to determine features with significant impact on fGOS and GOS0. The calculated weights have been reported in Table 3, Table 5, Table 8, and Table 10.

The limitations of our automated system are as follows:

1. Several missing data had to be omitted in this work.
2. Using our model for quick examinations of critical TBI patients is not flawless. Hence, our model needs to be validated using huge databases collected from different ethnicity before deploying for healthcare services.

## Conclusion

In this work, we have used ML methods such as RF and GLM for survival prediction of TBI patients in short- and long-term periods. However, significant development must be made before ML methods get ready for deployment in safety–critical applications such as medical diagnosis. According to our findings, the condition of pupils, GCSM, condition of cisterns, and the patients’ age are the best predictors of their survival.

As future work, the investigated models must be further evaluated. To this end, we plan to prepare larger and more versatile datasets from multiple medical centers. Having access to larger datasets leads to more robust model training and reliable evaluation. While we only focused on the mortality rate of TBI patients, investigating patients’ conditions after a predefined amount of time is worthy of future research.

## Data Availability

The datasets used and analysed during the current study are accessible by requesting the corresponding author.
